# Linkage mapping and expression analysis of miRNAs and their target genes during fiber development in cotton

**DOI:** 10.1186/1471-2164-14-706

**Published:** 2013-10-16

**Authors:** Xuemei Chen, Wenhui Gao, Jinfa Zhang, Xianlong Zhang, Zhongxu Lin

**Affiliations:** 1 National Key Laboratory of Crop Genetic Improvement & National Centre of Plant Gene Research (Wuhan), Huazhong Agricultural University, 430070 Wuhan, Hubei, China; 2 Department of Plant and Environmental Sciences, New Mexico State University, Box 30003, Las Cruces 88003, NM, USA

**Keywords:** Cotton, miRNA, Target genes, Chromosome mapping, RT-PCR

## Abstract

**Background:**

MicroRNAs (miRNAs) are small, endogenously expressed, non-coding RNA molecules involved in gene transcription and expression that combine with specific mRNA site of target genes to inhibit protein synthesis or degrade mRNA. Since the first plant miRNA was reported in 2002, numerous new miRNAs and their targets have been discovered via high-throughput sequencing and computational approaches. However, the genetic variation of miRNA genes is poorly understood due to the lack of miRNA-specific DNA markers.

**Results:**

To study the genetic variation and map miRNAs and their putative target genes in cotton, we designed specific primers based on pre-miRNAs and published putative target genes. A total of 83 pre-miRNA primers and 1,255 putative target gene primers were surveyed, and 9 pre-miRNA polymorphic loci were mapped on 7 of the 26 tetraploid cotton chromosomes. Furthermore, 156 polymorphic loci of the target genes were mapped on the cotton genome. To map more miRNA loci, miRNA-based SRAP (sequence-related amplified polymorphism) markers were used to map an additional 54 polymorphic loci on the cotton genome with the exception of Chr01, Chr22, and Chr24. Finally, a network between miRNAs and their targets was constructed. All pre-miRNAs and 98 putative target genes were selected for RT-PCR analysis, revealing unique expression patterns across different fiber development stages between the mapping parents.

**Conclusions:**

Our data provide an overview of miRNAs, their putative targets, and their network in cotton as well as comparative expression analyses between *Gossypium hirsutum* and *G. barbadense*. These data provide a foundation for understanding miRNA regulation during cotton fiber development.

## Background

MicroRNAs (miRNAs) are a class of non-coding small RNA molecules, generally 21 nucleotides (nt) in length that regulate critical functions in plant and animal development. miRNAs arise from an ~70-nt stem-loop RNA precursor that is cleaved and modified by Ribonuclease III to create an ~21-nt single-stranded miRNA [1,2]. Generally, every mature miRNA derives from one of the precursor’s arms and all are located in intergenic regions, indicating that miRNA transcription is independent of other genes and has its own transcriptional regulatory mechanisms. miRNAs regulate gene expression at the post-transcriptional level through mRNA cleavage or translational inhibition in both animals and plants [3-7]. In plant cells, the miRNA sequence is almost completely complementary with target gene mRNA, through a mechanism similar to RNA interference (RNAi), leading to the degradation of target gene mRNA [8]. Recently, numerous miRNAs have been reported through cloning, high-throughput small RNA sequencing, and computational approaches based on sequence similarities and secondary structure predictions [4]. Because plant miRNAs are almost exactly complementary to their corresponding target sequences [5], target gene prediction is relatively straightforward and mainly through bioinformatics method.

In 1993, the first miRNA (*lin-4* gene) was reported in *Caenorhabditis elegance*[9]. Then, Reinhart et al. [10] found the second metachronous switch gene, *let-7,* in *C. elegans*. Recently, with bioinformatics, molecular cloning technology improvements, and the establishment of model species cDNA libraries, hundreds of small RNA molecules have been successively reported in *C. elegans*[11,12], Arabidopsis [13,14], rice [15], maize [16], wheat [17], moss [18] and in cotton [19-22]. Almost 3,600 miRNAs have been annotated in Magnoliophyta including 291 from *Arabidopsis thaliana* and 40 from *Gossypium* species, as of April 21, 2012 (http://www.mirbase.org/cgi-bin/browse.pl). Recently, with the sequencing of the *G. raimondii* (D_5_) genome, many miRNA precursors and miRNAs were identified [23-25]. However, their distribution on the tetraploid cotton genome is unknown. To date, few miRNAs have been functionally characterized in cotton, and almost no predicted target genes have been verified by experiments in cotton.

Cotton is not only a fundamental world commodity that provides an important natural material, it is also an important oil crop. Since the first miRNA was reported, studies suggest that plant miRNAs negatively regulate target genes involved in plant development, organ morphogenesis, auxin signaling, and environmental stress responses [14,26-30]. MiRNAs may also play an important role in cotton fiber development and the study of miRNAs has far-reaching significance to improve fiber quality and yield [31].

At this time, most miRNAs and target gene research is focused on functional verification through gene over-expression, gene interference, and related methods, but there is paucity in development of miRNA markers and their relationship with phenotypes. Only one report in the literature described miRNA markers- miRNA-AFLP (amplified fragment length polymorphism) [32], but no miRNAs or their target genes have been genetically mapped in cotton.

To address this gap and to map miRNAs to understand their genomic distribution in cotton, we downloaded pre-miRNA sequences reported in the literature and within the miRBase database (Additional file 1: Table S1). Putative target genes were predicted by psRNATarget (http://plantgrn.noble.org/psRNATarget). Specific primers were then designed based on the pre-miRNA sequences and the target sites of the target genes, and miRNA-based SRAP marker analysis was performed. Primers were also designed surrounding the target sites in the putative target genes to indicate their chromosomal distribution. Then, a network diagram between miRNAs and their putative targets was constructed to reveal regulatory relationships. Finally, RT-PCR analysis was conducted to detect expression differences between *G. hirsutum* and *G. barbadense* in selected pre-miRNAs and putative target genes.

## Results and discussion

### Strategies of development markers for miRNAs and their target genes

Primers were designed for 123 pre-miRNA sequences and identical primers were eliminated with a nucleotide BLAST search. Finally, 83 pairs of pre-miRNA primers were obtained (Additional file 2: Table S2). Then, these primers were screened using single-stranded conformation polymorphism (SSCP), of which 12 primers yielding 13 polymorphic loci. The rate of primer polymorphism was 14.5%.

A total of 1,255 primer pairs were obtained for the 1,399 target genes of the miRNAs after eliminating identical primers as described above (Additional file 3: Table S3). Of these, 147 primer pairs produced 161 polymorphic loci after SSCP analysis. The rate of primer polymorphism was 11.6%.

Based on miRNA-AFLP technology, the miRNA-SRAP marker technique was performed to enrich miRNA markers. Among the 2,944 miRNA-SRAP primer combinations, ~8 scorable bands per primer combination were typically produced. Exceptions were miR319-SRAP primer combinations which generated only 0–2 scorable bands. These results are consistent with those obtained from miRNA-based AFLP marker techniques [32]. Fifty-five primers produced 59 polymorphic loci (Additional file 4: Table S4), and the rate of primer polymorphism was 1.87%. No polymorphic amplification products were obtained for miR156, miR319, miR390, miR398, and miR399 when combined with SRAP primers.

Pang et al. [32] explored the possibility of using miRNA genes as markers and identified DNA sequences of potential pre-miRNA. miRNA-AFLP analysis provides a reliable targeted genotyping strategy to assess genetic diversity among cotton species. The SRAP primers can target exons and introns respectively and generate polymorphism based them [33]; they also can combine with primers desighed from candidate gene regions, which is also called TRAP (Target Region Amplification Polymorphism) [34]. Therefore, when primers designed from conservative miRNAs and their complementary sequences combining with SRAP primers, miRNAs or their flanking sequences can be amplified. This is a highly effective strategy to examine miRNA gene distribution as well as more feasible, simple, and efficient than the miRNA-AFLP technique with respect to typing miRNA markers. Also, miRNA-SRAP markers may be functional genes; therefore, the miRNA-SRAP technique has important significance to the study of miRNAs.

### Verification of miRNA-SRAP marker techniques

**Figure 1:**
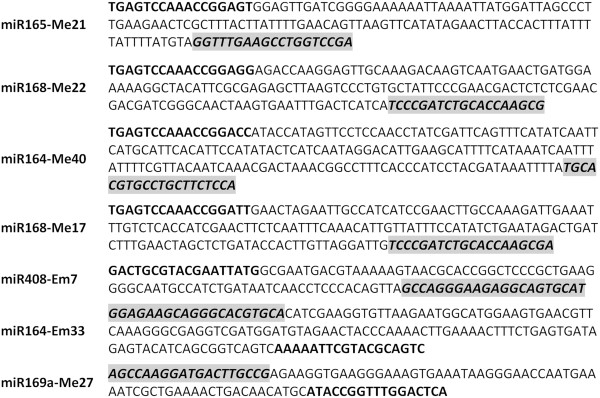
**Verification of miRNA-based SRAP marker technology by sequencing.** SRAP primers are in bold. miRNA primers are in Italic, bolded, and in shadow.

To verify whether the miRNA-SRAP marker technique reliably amplified miRNA sequences, PCR products were cloned and sequenced from a sample of 7 random polymorphic miRNA-SRAP primer combinations. Then, miRNA primers and SRAP primers were identified as depicted in Figure 1. The figure depicts reliably amplified products using the primer combinations instead of amplified products from individual miRNA primers or SRAP primers.

### Even distribution of miRNA markers and preferential distribution of target gene markers in the cotton genome

After linkage analysis, 9 of the 13 polymorphic loci of pre-miRNAs were mapped on 7 cotton chromosomes (Chr01, Chr04, Chr13, Chr15, Chr16, Chr23 and Chr26) based on SSCP analysis which can discover minor sequence mutations and reveal more polymorphisms to map genes [35]. For miRNA-based SRAP markers, 54 of the 59 polymorphic loci were mapped on 23 cotton chromosomes, except for Chr01, Chr22, and Chr24. These data suggest that miRNA markers are widely distributed on 24 chromosomes, except for Chr22 and Chr24, for which no miRNA markers were identified. Apparently, relatively more loci occurred on Chr10 (5 loci) and Chr19 (6 loci). Thirty-two loci were mapped on 13 chromosomes of the At sub-genome, and 31 loci were mapped on 11 chromosomes of the Dt sub-genome (Additional file 5: Figure S1, Additional file 6: Table S5). In conclusion, these 63 miRNA loci were evenly distributed on 24 chromosomes, except Chr22 and Chr24.

Of the 161 polymorphic loci for the putative miRNA target genes, 156 were mapped on the cotton genome: 59 loci were mapped on 13 chromosomes of the At sub-genome, and 97 loci were mapped on 13 chromosomes of the Dt sub-genome (Additional file 5: Figure S1, Additional file 6: Table S5). Overall, unlike miRNA markers, target gene markers were preferentially distributed on the Dt sub-genome. Specifically, chromosomes Chr13, Chr18, Chr19 and Chr21 contained a total of 47 target gene marker loci, i.e., 30% of the total target gene marker loci. Comparatively fewer less loci were on Chr01, Chr03, Chr06, Chr07, Chr08 and Chr09. Surprisingly, 95 of the 156 target gene polymorphic loci belonged to target genes from the miR414 family, whereas 61 loci belonged to 26 other miRNAs families.

To date, no miRNA genes and their target genes have been genetically mapped in cotton. Pang et al. [32] developed conserved miRNA gene markers and explored the possibility of an miRNA-AFLP marker technique. Then, the amplified products were sequenced and compared for homology using cotton expressed sequence tags. However, genetic mapping of miRNA markers has not been reported. Thus, miRNAs and their target gene markers reported here were genetically mapped in our interspecific *G. hirsutum* × *G. barbadense* population. Such genetic mapping of a segregating population provided an overview of the chromosomal distribution of miRNAs and their target genes in cotton.

### Network diagram revealed by relationships between miRNAs and their target genes

**Figure 2:**
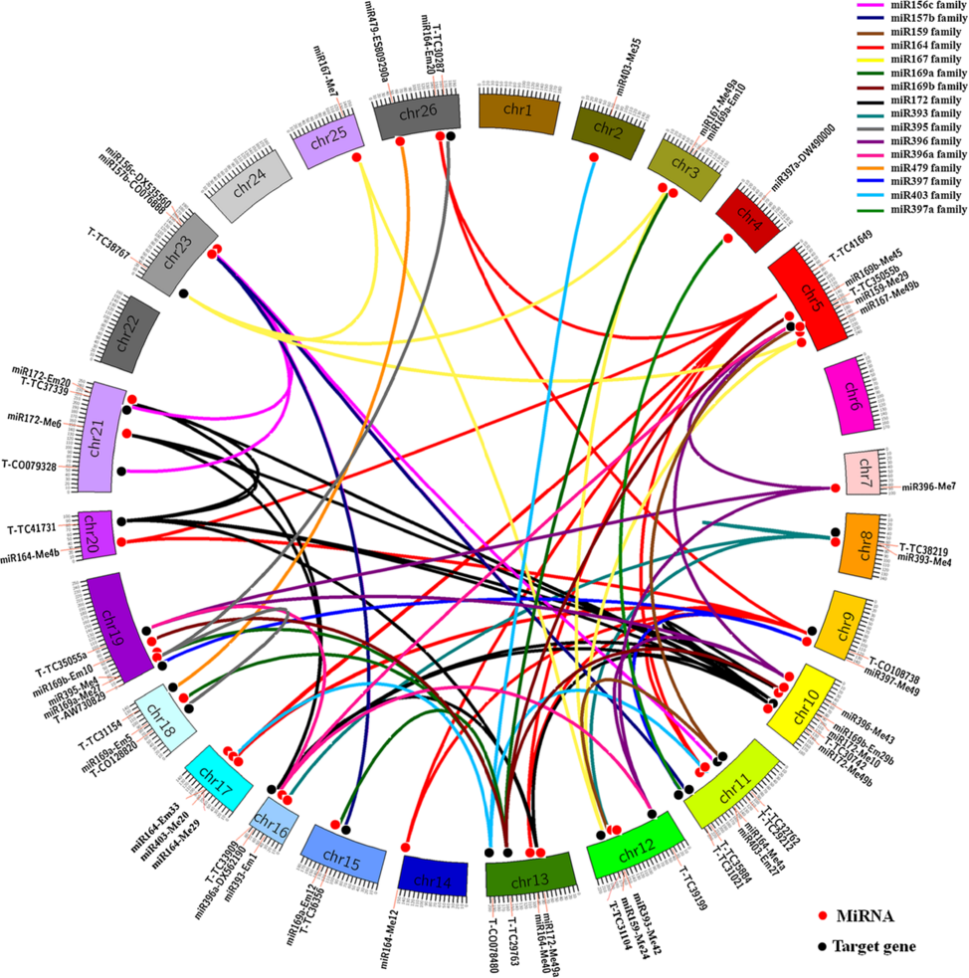
**Network diagram revealed by relationships between miRNA families and their target genes.** Red circle: MiRNAs; black circle: Target genes. The scale marked on each chromosome represents genetic map distance (cM). miRNA primers and target primers are marked on the chromosome, and the miRNA family and target gene relationships are indicted by each color.

In order to excavate the valuable miRNAs and putative targets for further research, miRNAs and putative target genes, mapped on the interspecific BC_1_ genetic linkage map, were used to create a network diagram based on their regulatory relationships (Figure 2, Table 1) by Circos (http://circos.ca/). Table 1 depicts 39 miRNAs and 24 target genes that are contained in this network diagram. Some miRNAs were associated with more than one target gene, and some target genes were regulated by more than one miRNA family. Individual miRNAs and their target genes are clearly and intuitively depicted in the network diagram (Figure 2, Additional file 7: Figure S2). As shown in Additional file 7: Figure S2, miR393 and its target TC38219 (Additional file 7: Figure S2I), and miR172 and its target TC30742 were located on the same chromosome (Additional file 7: Figure S2H). Also, miR393 and its target TC38219 were proximal on the same chromosome, separated only by 1.78 cM; whereas, miR172-Me10 and its target TC30742 were separated by 30.72 cM. miR172-Me49b and its target TC30742 were separated by 11.41 cM (Table 1). Additional file 7H shows that miR172 and its target TC30742 (both located on Chr10), and another target TC41731 (located on Chr20), are found on homologous chromosomes (Chr10 and Chr20). The miR396 (Additional file 7: Figure S2K) and miR396a families (Additional file 7: Figure S2L) have the same targets as depicted in the network diagram: TC35055 and TC39199. Chromosomes on which miR396 and miR396a are located are also on homologous chromosomes (Chr07 and Chr16), and their target, TC35055, has two sites. Furthermore, TC350055a, located on Chr19, and TC350055b, located on Chr05, are found on homologous chromosomes. Additional file 7: Figure S2G depicts the same information: miR196b-Me45 was located on Chr05 and miR196b-Em10 was located on Chr19. For miR156c-DX53560 (Additional file 7: Figure S2A), its target, TC37339 was located on Chr21 and another target TC29212 was located on Chr11, which are homologous chromosomes. Additional file 7: Figure S2F depicts the location of miR169a (Chr18) and TC29763 (Chr13), which are also located on homologous chromosomes. Other miRNAs and their target genes were located on different chromosomes.

**Table 1:** **miRNAs and their target genes localization on the interspecific BC**_**1 **_**linkage map**

**miRNAs name**	**Chromosome**	**Subgenome**	**Genetic map**	**Target name**	**Chromosome**	**Subgenome**	**Genetic map**
			**distance (cM)**				**distance (cM)**
miR156c-DX535560★	Chr23	D	154.258	T-TC29212	Chr11	A	112.712
				T-CO079328	Chr21	D	56.729
				T-TC37339	Chr21	D	220.676
miR156c-DX535560★	Chr23	D	154.258	T-TC35884▲	Chr11	A	214.024
				T-TC36356▲	Chr15	D	118.218
miR157b-CO076888★	Chr23	D	150.354	T-TC35884▲	Chr11	A	214.024
				T-TC36356▲	Chr15	D	118.218
miR159-Me29	Chr05	A	165.2	T-TC32762	Chr11	A	94.707
miR159-Me24	Chr12	A	156.471	T-TC32762	Chr11	A	94.707
miR164-Me4a★	Chr11	A	146.281	T-TC41649	Chr05	A	70.705
miR164-Me4b★	Chr20	D	26.305	T-TC41649	Chr05	A	70.705
				T-CO108738	Chr09	A	124.811
miR164-Me12★	Chr14	D	157.964	T-TC41649	Chr05	A	70.705
				T-CO108738	Chr09	A	124.811
miR164-Me29★	Chr17	D	79.157	T-TC41649	Chr05	A	70.705
				T-CO108738	Chr09	A	124.811
miR164-Me40★	Chr13	A	91.878	T-TC41649	Chr05	A	70.705
				T-CO108738	Chr09	A	124.811
miR164-Em33★	Chr17	D	82.728	T-TC41649	Chr05	A	70.705
				T-CO108738	Chr09	A	124.811
miR164-Em20★	Chr26	D	143.941	T-TC41649	Chr05	A	70.705
				T-CO108738	Chr09	A	124.811
miR167-Me7★	Chr25	D	126.296	T-TC31104	Chr12	A	169.342
				T-TC38767	Chr23	D	25.125
miR167-Me49a★	Chr03	A	67.893	T-TC31104	Chr12	A	169.342
				T-TC38767	Chr23	D	25.125
miR167-Me49b★	Chr05	A	198.84	T-TC31104	Chr12	A	169.342
				T-TC38767	Chr23	D	25.125
miR169a-Me27	Chr19	D	18.195	T-TC29763▲	Chr13	A	153.525
miR169a-Em5	Chr18	D	84.73	T-TC29763▲	Chr13	A	153.525
miR169a-Em10	Chr03	A	75.625	T-TC29763▲	Chr13	A	153.525
miR169a-Em12	Chr15	D	134.58	T-TC29763▲	Chr13	A	153.525
miR169b-Me45	Chr05	A	131.477	T-TC29763▲	Chr13	A	153.525
miR169b-Em10	Chr19	D	64.043	T-TC29763▲	Chr13	A	153.525
miR169b-Em29b	Chr10	A	87.599	T-TC29763▲	Chr13	A	153.525
miR172-Me6★	Chr21	D	149.272	T-TC30742	Chr10	A	123.541
				T-TC33909	Chr16	D	82.967
				T-TC41731	Chr20	D	80.438
miR172-Me10★	Chr10	A	92.823	T-TC30742	Chr10	A	123.541
				T-TC33909	Chr16	D	82.967
				T-TC41731	Chr20	D	80.438
miR172-Me49a★	Chr13	A	76.047	T-TC30742	Chr10	A	123.541
				T-TC33909	Chr16	D	82.967
				T-TC41731	Chr20	D	80.438
miR172-Me49b★	Chr10	A	134.949	T-TC30742	Chr10	A	123.541
				T-TC33909	Chr16	D	82.967
				T-TC41731	Chr20	D	80.438
miR172-Em20★	Chr21	D	229.243	T-TC30742	Chr10	A	123.541
				T-TC33909	Chr16	D	82.967
				T-TC41731	Chr20	D	80.438
miR393-Me4	Chr08	A	67.257	T-TC38219	Chr08	A	65.478
miR393-Me42	Chr12	A	154.801	T-TC38219	Chr08	A	65.478
miR393-Em1	Chr16	D	44.796	T-TC38219	Chr08	A	65.478
miR395-Me4★	Chr19	D	18.742	T-CO128820	Chr18	D	65.452
				T-TC30287	Chr26	D	162.675
miR396-Me7★	Chr07	A	85.357	T-TC35055b▲	Chr05	A	155.907
				T-TC39199▲	Chr12	A	35.006
				T-TC35055a▲	Chr19	D	95.301
miR396-Me43★	Chr10	A	53.687	T-TC35055b▲	Chr05	A	155.907
				T-TC39199▲	Chr12	A	35.006
				T-TC35055a▲	Chr19	D	95.301
miR396a-DX562190★	Chr16	D	73.216	T-TC35055b▲	Chr05	A	155.907
				T-TC39199▲	Chr12	A	35.006
				T-TC35055a▲	Chr19	D	95.301
miR397-Me49★	Chr09	A	145.396	T-AW730829	Chr11	A	226.873
				T-TC31021▲	Chr19	D	0
miR397a-DW490000★	Chr04	A	59.354	T-TC31021▲	Chr19	D	0
miR403-Me35	Chr02	A	71.74	T-CO078480	Chr13	A	194.48
miR403-Em27	Chr11	A	146.949	T-CO078480	Chr13	A	194.48
miR403-Me20	Chr17	D	84.782	T-CO078480	Chr13	A	194.48
miR479-ES809290a	Chr26	D	44.775	T-TC31154	Chr18	D	121.25

★: miRNA was associated with more than one target on the interspecific BC_1_ linkage map.

▲: Target was regulated by more than one miRNAs family on the interspecific BC_1_ linkage map.

In our network diagram, the relationship and distribution of miRNAs and their putative target genes on the interspecific BC_1_ genetic linkage map are clearly and intuitively revealed. This diagram will facilitate our understanding of the regulatory mechanisms and networks for target genes and cell development in cotton. Allen et al. [36] stated that plant miRNAs are thought to be derived from their target sequences after gene duplication, inverted duplication, and divergence in miRNA evolution. Additional file 7: Figure S2 shows that many miRNAs and their targets, miRNAs with the same target, and targets of the same miRNA family were located on homologous chromosomes or the same chromosome. These data are consistent with Allen’s published findings [36]. Other miRNAs and their targets did not reside on the same or homologous chromosomes, which may be due to evolutionary mutations or deletions or insufficient marker numbers. It was noteworthy that homologous chromosomes, Chr5 and Chr19, contained more targets and mirRNAs as depicted in the network diagram, and this may have implications for future studies of miRNAs and their targets.

### Expression differences between parents by RT-PCR and qRT-PCR

**Figure 3:**
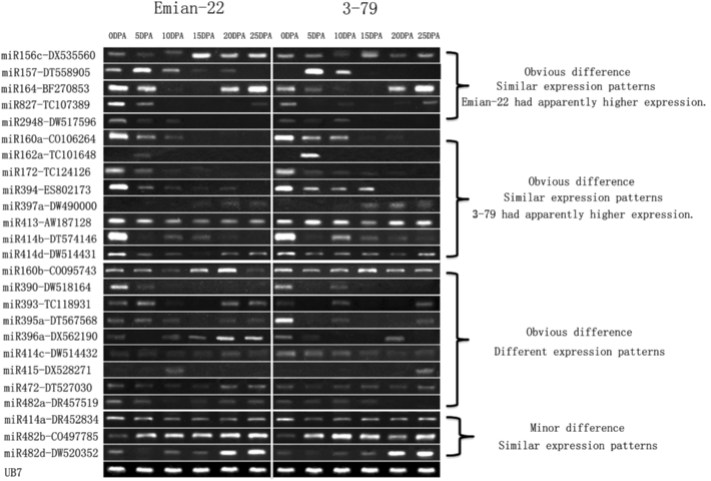
**RT-PCR analysis of pre-miRNAs between mapping parents.** Numbers at the top represent 0DPA, 5DPA, 10DPA, 15DPA, 20DPA and 25DPA of Emian22 and 3–79, respectively. Similar expression tendencies between Emian22 and 3–79 were classified into similar expression patterns. Obvious differential expression tendencies between Emian22 and 3–79 were classified into differential expression patterns. Obvious differential expression levels between Emian22 and 3–79 were classified into obvious difference. Minor differential expression levels between Emian22 and 3–79 were classified accordingly. Gene primers are labeled on the left.

We used RT-PCR analysis to investigate pre-miRNAs and their putative target genes during fiber development between *G. hirsutum* and *G. barbadense*. A total of 181 primer pairs were used for RT-PCR analysis, including 83 pre-miRNA primers and 98 randomly selected target gene primers. Twenty-five pre-miRNA genes (30.1%) were expressed in cotton fibers. Among them, 88% of these expressed pre-miRNAs were significantly different among various stages of fiber development (0, 5, 10, 15, 20, and 25 DPA) between Emian22 and 3–79. Ten pre-miRNAs had weak or no expression in both Emian22 and 3–79 (defined as no expression differences in this study). Furthermore, 13 pre-miRNAs had similar expression patterns, and 9 pre-miRNAs had different expression patterns (Figure 3). Figure 3 also shows that 40% of the pre-miRNA genes had higher expression in early ovary development.

**Figure 4:**
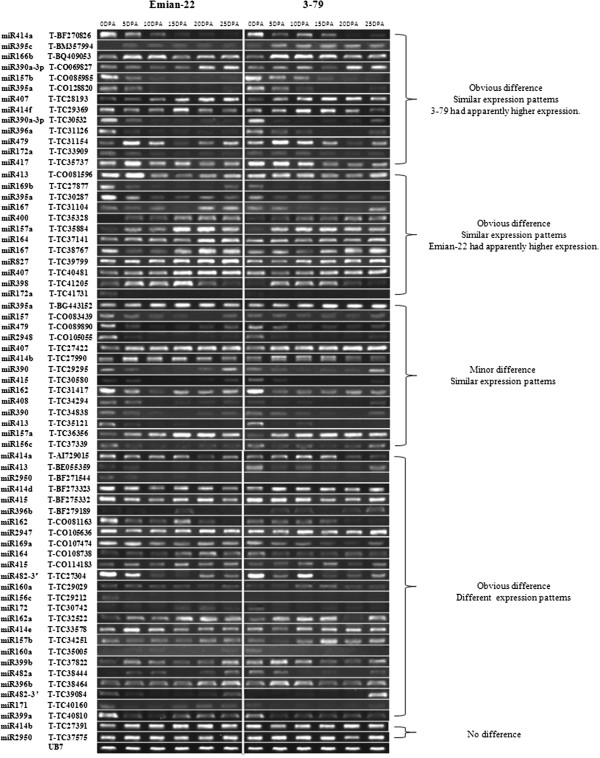
**RT-PCR analysis of target genes between mapping parents.** Numbers at the top represent 0DPA, 5DPA, 10DPA, 15DPA, 20DPA and 25DPA of Emian22 and 3–79, respectively. Similar expression tendencies between Emian22 and 3–79 were classified into similar expression patterns. Obvious differential expression tendencies between Emian22 and 3–79 were classified into differential expression patterns. Obvious differential expression levels between Emian22 and 3–79 were classified into obvious difference. Minor differential expression levels between Emian22 and 3–79 were classified accordingly. Gene primers and their corresponding miRNA families are labeled on the left, and only one miRNA family is listed. More family information was shown in Additional file 8.

Regarding target genes, 66 (67.3%) were expressed in cotton fiber (Additional file 8: Table S6). Among them, 50 were obviously differentially expressed; 14 had minor differences; and 2 were not different (Figure 4). Overall, 75.8% of the expressed target genes were significantly different at various fiber development stages (0, 5, 10, 15, 20, and 25 DPA), when comparing Emian22 and 3–79. Three target genes were weakly expressed or not expressed at all stages in both Emian22 and 3–79 (defined as no expression differences in this study). Furthermore, 39 target genes had similar expression patterns. Of the 25 target genes with different expression patterns, 13 were up-regulated and 12 were down regulated in 3–79, compared with Emian22. To further confirm RT-PCR data, target genes belonging to different categories were randomly chosen for qRT-PCR analysis (Additional file 9: Figure S3). Consistent results were observed between both RT-PCR and qRT-PCR analyses.

In this study, a diagram was constructed to depict pre-miRNAs and their corresponding target genes (Additional file 10: Figure S4). Additional file 10: Figure S4 shows that miR156c-DX535560, miR472-DT527030, and miR482a-DR457519 were similar with respect to their targets in various fiber development stages of Emian22. However, no correlation was observed between pre-miRNA and its target expression in various fiber development stages of 3–79. MiR157-DT558905, miR395a-DT567568 and miR395a-DT567568 had contrasting expression tendencies with their targets in various stages of Emian22, but no correlation was observed between the expression of pre-miRNA and its targets in various fiber development stages of 3–79. MiR172-TC124126, miR413-AW187128, and miR414a-DR452834 and their targets had similar expression tendencies in various parent stages. MiR414d-DW514431 had contrasting expression tendencies with its targets in various stages of Emian22 and had similar expression tendencies in various stages of 3–79.

In summary, we report dynamic expression of pre-miRNAs and putative target genes at various fiber development stages in *G. hirsutum* and *G. barbadense*. Most expressed pre-miRNAs (88%) and their putative targets (75.8%) were obviously different with respect to expressions at different stages between the two parents. Unique expression patterns of pre-miRNAs and their putative targets may be connected with a particular function. We also found that pre-miRNA families and their putative targets had correlated expression tendencies in various growth stages, suggesting that miRNA genes may regulate target gene expression. However, the biological relevance of this must be investigated further to better understand regulatory mechanisms and the overall network of plant growth control.

## Conclusions

In this study, miRNAs and target markers were developed. We found that although the primers were specific, pre-miRNA primer polymorphisms were low (14.5%), and target primer polymorphisms were even lower (11.7%). The 63 miRNA loci were evenly distributed on 24 chromosomes, except Chr22 and Chr24. Also, 156 target gene loci were preferentially distributed on the Dt sub-genome and some chromosomes. Genetic mapping in a segregating population provides an opportunity to examine distribution of miRNAs and their target genes. A network diagram depicting miRNAs and their targets confirmed that plant miRNAs may be derived from their target sequences after gene duplication or inverted duplication [36]. Comparative expression analyses of *Gossypium hirsutum* and *G. barbadense* revealed that miRNAs and their target genes play a role in cotton fiber development.

## Methods

### Marker development

**Figure 5:**
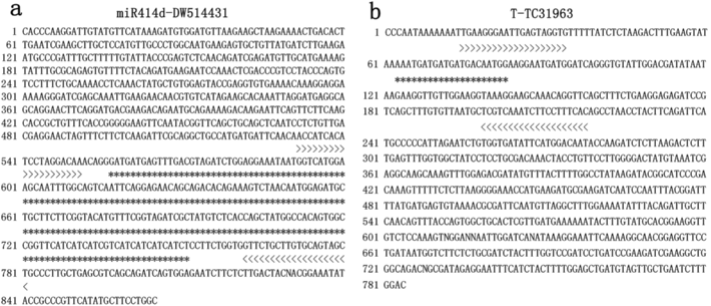
**Primer design strategies for miRNAs and target genes. (a)** miR414d from DW514431 (Genbank acc No. DW514431); **(b)** target gene of miR414d from TC31963 (Cotton Gene Index No. TC31963, http://compbio.dfci.harvard.edu/tgi/cgi-bin/tgi/gimain.pl?gudb=cotton). *: Target sequence; >>>>>: Primer region.

Pre-miRNA sequences reported in the literature and a database (http://www.mirbase.org) were downloaded [37-43] (Additional file 1: Table S1). Putative target genes were predicted and downloaded with psRNATarget (http://plantgrn.noble.org/psRNATarget/). Because one target gene may relate to more than one miRNA, repeat target sequences were deleted. Finally, a total of 123 pre-miRNA sequences and 1,399 putative target sequences were obtained. Primers were then designed based on the pre-miRNAs and the target sites of the putative target genes by Primer 3.0 (examples in Figure 5). Criteria for primer design were a primer length of 18–25 bp (20 bp optimum), GC content of 35–70% (50% optimum), annealing temperature of 50–65°C (55°C optimum), and PCR product size of 100–1,000 bp.

Because the polymorphism is low in cotton, to genetically map more miRNAs, SRAP primers, including 64 forward primers and 64 reverse primers [44] were combined with 23 miRNA degenerate primers [32] in the form of miRNA-SRAP analysis. Finally, a total of 2,944 primer combinations were applied in this study.

### PCR amplification and electrophoresis

PCR of pre-miRNAs and their putative target genes was performed in a solution (10 μL) containing 25 ng DNA template, 1 × Buffer, 2.0 mmol L^-1^ MgCl_2_, 0.25 mmol L^-1^ dNTPs, 0.16 μmol L^-1^ of forward primer, 0.16 μmol L^-1^ of reverse primer, 0.8 units of Taq DNA polymerase, and ddH_2_O was added to 10 μL. The PCR program was performed using the following profile: 95°C for 5 min, followed by 34 cycles consisting of 94°C for 50 sec, 56°C for 45 sec, and 72°C for 60 sec; and a final extension step of 5 min at 72°C. Then, PCR products were separated on an 8% non-denaturing polyacrylamide gel at a constant voltage (15 W) for about 4 h at room temperature. After electrophoresis, DNA fragments were detected by silver staining, coloring in a sodium hydroxide and formaldehyde solution.

PCR of an miRNA-based SRAP marker was performed in a solution (10 μL) containing 30 ng DNA template, 1 × Buffer, 2.0 mmol L^-1^ MgCl_2_, 0.2 mmol L^-1^ dNTPs, 0.1 μmol L^-1^ of forward primer, 0.1 μmol L^-1^ of reverse primer, 0.5 units of Taq DNA polymerase, and ddH_2_O was added to 10 μL. The PCR program was performed using the following profile: 94°C for 5 min, followed by 4 cycles of 94°C for 1 min, 35°C for 1 min, and 72°C for 1 min; then by 34 cycles of 94°C for 1 min, 50°C for 1 min, and 72°C for 1 min; and a final extension step of 10 min at 72°C. Then, PCR products were separated on a 6% denaturing polyacrylamide gel (29:1 acrylamide and N, N-methylene bisacrylamide) at a constant voltage (80 W) for about 2 h at room temperature. After electrophoresis, DNA fragments were detected by silver staining, coloring in a sodium hydroxide and formaldehyde solution.

### Genetic mapping

Our BC_1_ mapping population ([Emian22 × 3–79] × Emian22) [45,46] was used to map miRNAs and their putative target genes. All primers with polymorphisms between parents were used to enrich the interspecific BC_1_ genetic linkage map [46]. Polymorphic loci were integrated into the interspecific BC_1_ linkage map using JoinMap V3.0 [47]. The logarithm of odds (LOD) threshold was 5.0. Map distances in centi Morgans (cM) were calculated using the Kosambi mapping function [48]. The linkage map was drawn by MapChart V2.2 software [49].

### Drawing method of network diagram

In this study, miRNAs and their putative target genes were mapped in cotton chromosomes. The distribution of miRNAs and their putative target markers was obtained from the genetic linkage map. MiRNA loci and their putative target loci that were mapped on the chromosomes were then “connected” using individually colored lines to indicate each miRNA family’s connection to their putative target genes. Then, a network diagram was obtained based on the distribution of miRNAs and their target markers on genetic linkage map by Circos (http://circos.ca/).

### RT-PCR and qRT-PCR analysis

To determine the expression difference of pre-miRNAs and their putative target genes during fiber development between *G. hirsutum* and *G. barbadense*, RNAs were extracted from developing fibers at 0 days post anthesis (DPA), 5 DPA, 10 DPA, 15 DPA, 20 DPA and 25 DPA, and RNAs (4 μg) were reverse-transcribed to cDNA by the M-MLV-RT Reverse Transcriptase (Invitrogen). RT-PCR and qRT-PCR analyses were performed according to the methods described by Munis et al. [50] with minor modifications. Ubiquitin (GenBank acc No.: DQ116441) primer pair (forward 5′GAAGGCATTCCACCTGACCAAC3′, reverse 5′CTTGACCTTCTTCTTCTTGTGCTTG 3′) [51] was used as an internal standard showing the equal amounts of first-stranded cDNA in each sample. In addition, qRT-PCR experiments were biologically repeated three times.

## Competing interests

The authors declare that they have no competing interests.

## Authors’ contributions

XC designed the primers, performed the experiments, and drafted the manuscript. WG made the network diagram. JZ provided the miRNA degenerate primers. XZ participated in study design and manuscript proofreading. ZL directed the experiments, proofread the manuscript, and advised the scientific writing. All authors contributed to data interpretation, read, and approved the final manuscript.

## Supplementary Material

Additional file 1: Table S1 Source of miRNA sequences and distribution in the scientific literature. Click here for file

Additional file 2: Table S2 Characteristics of 83 pre-miRNA primers in this study. √: Polymorphic primers; ×: No polymorphic primers: The primer was not mapped on the chromosome. Click here for file

Additional file 3: Table S3 Characteristics of 1,255 target primers in this study. √: Polymorphic primers; ×: No polymorphic primers. -: The primer was not mapped on the chromosome. Click here for file

Additional file 4: Table S4 Characteristics of 55 miRNA-SRAP polymorphic primer combinations in this study-: The primer was not mapped on the chromosome. Click here for file

Additional file 5: Figure S1 Locations of polymorphic markers on the BC_1_ genetic linkage map. miRNA markers are underlined and in bold. Target markers are underlined. miRNA-based SRAP markers are italic and in bold. Click here for file

Additional file 6: Table S5 Marker distribution on the interspecific BC_1_ genetic linkage map. Click here for file

Additional file 7: Figure S2 Network diagram revealed by relationships between one miRNA family and their target genes. Families are as follows: A: miR156c family; B: miR157b family; C: miR159 family; D: miR164 family; E: miR167 family; F: miR169a family; G: miR169b family; H: miR172 family; I: miR393 family; J: miR395 family; K: miR396 family; L: miR396a family; M: miR397 family; N: miR397a family; O: miR403 family; P: miR479 family. Red circle: MiRNAs; black circle: Target genes. The scale marked on each chromosome represented a genetic map distance (cM). Click here for file

Additional file 8: Table S6 Target gene primers and their corresponding miRNA families. Click here for file

Additional file 9: Figure S3 qRT-PCR analysis of eight primers randomly chosen from pre-miRNAs and targets. Expression levels of Emian22 and 3–79 are shown. “*” represents P⩽0.05, and “**” represents P⩽0.01. Primers are as follows: A: miR164-BF270853; B: miR393-TC118931; C: miR413-AW187128; D: T-BF275332; E: T-BM3579942; F: T-TC27422; G: T-TC37575; H: T-TC38767. Click here for file

Additional file 10: Figure S4 Wiring diagram of target gene primers and their corresponding miRNA families. (a) RT-PCR analysis of miRNA genes; (b) RT-PCR analysis of target genes. Click here for file

## References

[B1] Reinhart BJ Weinstein EG Rhoades MW Bartel B Bartel DP MicroRNAs in plants Genes Dev 2002 14 17 2313 2313 10.1101/gad.1004402 PMC186362 12101121

[B2] Lai EC MicroRNAs: runts of the genome assert themselves Curr Biol 2003 14 23 R925 R936 10.1016/j.cub.2003.11.017 14654021

[B3] Carrington JC Ambros V Role of microRNAs in plant and animal development Science 2003 14 5631 336 338 10.1126/science.1085242 12869753

[B4] Ambros V Bartel B Bartel DP Burge CB Carrington JC Chen XM Dreyfuss G Eddy SR Griffiths-Jones S Marshall M Ruvkun G Tuschl T A uniform system for microRNA annotation RNA-Publ RNA Soc 2003 14 3 277 279 10.1261/rna.2183803 PMC1370393 12592000

[B5] Bartel DP MicroRNAs: genomics, biogenesis, mechanism, and function Cell 2004 14 2 281 297 10.1016/S0092-8674(04)00045-5 14744438

[B6] Yu ZR Raabe T Hecht NB MicroRNA Mirn122a reduces expression of the posttranscriptionally regulated germ cell transition protein 2 (Tnp2) messenger RNA (mRNA) by mRNA cleavage Biol Reprod 2005 14 3 427 433 10.1095/biolreprod.105.040998 15901636

[B7] Bushati N Cohen SM MicroRNA functions Annu Rev Cell Dev Biol 2007 14 175 205 10.1146/annurev.cellbio.23.090506.123406 17506695

[B8] Jones-Rhoades MW Bartel DP Bartel B MicroRNAs and their regulatory roles in plants Annu Rev Plant Biol 2006 14 19 53 10.1146/annurev.arplant.57.032905.105218 16669754

[B9] Lee RC Feinbaum RL Ambros V The *C. elegans* heterochronic gene *lin-4* encodes small RNAs with antisense complementarity to *lin-14* Cell 1993 14 5 843 854 10.1016/0092-8674(93)90529-Y 8252621

[B10] Reinhart BJ Slack FJ Basson M Pasquinelli AE Bettinger JC Rougvie AE Horvitz HR Ruvkun G The 21-nucleotide let-7 RNA regulates developmental timing in *Caenorhabditis elegans* Nature 2000 14 6772 901 906 10.1038/35002607 10706289

[B11] Lau NC Lim LP Weinstein EG Bartel DP An abundant class of tiny RNAs with probable regulatory roles in *Caenorhabditis elegans* Science 2001 14 5543 858 862 10.1126/science.1065062 11679671

[B12] Lee RC Ambros V An extensive class of small RNAs in *Caenorhabditis elegans* Science 2001 14 5543 862 864 10.1126/science.1065329 11679672

[B13] Llave C Xie ZX Kasschau KD Carrington JC Cleavage of scarecrow-like mRNA targets directed by a class of *Arabidopsis* miRNA Science 2002 14 5589 2053 2056 10.1126/science.1076311 12242443

[B14] Sunkar R Zhu JK Novel and stress-regulated microRNAs and other small RNAs from *Arabidopsis* Plant cell 2004 14 8 2001 2019 10.1105/tpc.104.022830 15258262 PMC519194

[B15] Sunkar R Girke T Jain PK Zhu JK Cloning and characterization of MicroRNAs from rice Plant cell 2005 14 5 1397 1411 10.1105/tpc.105.031682 15805478 PMC1091763

[B16] Mica E Gianfranceschi L Pe ME Characterization of five microRNA families in maize J Exp Bot 2006 14 11 2601 2612 10.1093/jxb/erl013 16820394

[B17] Yao YY Guo GG Ni ZF Sunkar R Du JK Zhu JK Sun QX Cloning and characterization of microRNAs from wheat (*Triticum aestivum* L) Genome Biol 2007 14 R96 10.1186/gb-2007-8-6-r96 17543110 PMC2394755

[B18] Arazi T Talmor-Neiman M Stav R Riese M Huijser P Baulcombe DC Cloning and characterization of micro-RNAs from moss Plant J 2005 14 6 837 848 10.1111/j.1365-313X.2005.02499.x 16146523

[B19] Abdurakhmonov IY Devor EJ Buriev ZT Huang L Makamov A Shermatov SE Bozorov T Kushanov FN Mavlonov GT Abdukarimov A Small RNA regulation of ovule development in the cotton plant, G. *hirsutum* L BMC Plant Biol 2008 14 93 10.1186/1471-2229-8-93 18793449 PMC2564936

[B20] Yu N Cai WJ Wang S Shan CM Wang LJ Chen XY Temporal control of trichome distribution by microRNA156-targeted SPL genes in *Arabidopsis thaliana* Plant Cell 2010 14 7 2322 2335 10.1105/tpc.109.072579 20622149 PMC2929091

[B21] Romanel E Silva T Corrêa R Farinelli L Hawkins J Schrago CG Vaslin MS Global alteration of microRNAs and transposon-derived small RNAs in cotton (*Gossypium hirsutum*) during Cotton leafroll dwarf polerovirus (CLRDV) infection Plant Mol Biol 2012 14 1 18 10.1007/s11103-012-9947-5 22987114

[B22] Wei M Wei H Wu M Song M Zhang J Yu J Fan S Yu S Comparative expression profiling of miRNA during anther development in genetic male sterile and wild type cotton BMC Plant Biol 2013 14 1 66 10.1186/1471-2229-13-66 23597285 PMC3639194

[B23] Paterson AH Wendel JF Gundlach H Guo H Jenkins J Jin D Llewellyn D Showmaker KC Shu S Udall J Yoo MJ Byers R Chen W Doron-Faigenboim A Duke MV Gong L Grimwood J Grover C Grupp K Hu G Lee TH Li J Lin L Liu T Marler BS Page JT Roberts AW Romanel E Sanders WS Szadkowski E Tan X Tang H Xu C Wang J Wang Z Zhang D Zhang L Ashrafi H Bedon F Bowers JE Brubaker CL Chee PW Das S Gingle AR Haigler CH Harker D Hoffmann LV Hovav R Jones DC Lemke C Mansoor S ur Rahman M Rainville LN Rambani A Reddy UK Rong JK Saranga Y Scheffler BE Scheffler JA Stelly DM Triplett BA Van Deynze A Vaslin MF Waghmare VN Walford SA Wright RJ Zaki EA Zhang T Dennis ES Mayer KF Peterson DG Rokhsar DS Wang X Schmutz J Repeated polyploidization of *Gossypium* genomes and the evolution of spinnable cotton fibres Nature 2012 14 7429 423 427 10.1038/nature11798 23257886

[B24] Wang KB Wang ZW Li FG Ye WW Wang JY Song GL Yue Z Cong L Shang HH Zhu SL Zou CS Li Q Yuan YL Lu CR Wei HL Gou CY Zheng ZQ Yin Y Zhang XY Liu K Wang B Song C Shi N Kohel RJ Percy RG Yu JZ Zhu YX Yu SX The draft genome of a diploid cotton *Gossypium raimondii* Nat Genet 2012 14 10 1098 1103 10.1038/ng.2371 22922876

[B25] Li Q Jin X Zhu YX Identification and analyses of miRNA genes in allotetraploid *Gossypium hirsutum* fiber cells based on the sequenced diploid *G. raimondii* genome J Genet Genomics 2012 14 7 351 360 10.1016/j.jgg.2012.04.008 22835981

[B26] Emery JF Floyd SK Alvarez J Eshed Y Hawker NP Izhaki A Baum SF Bowman JL Radial patterning of *Arabidopsis* shoots by class IIIHD-ZIP and KANADI genes Curr Biol 2003 14 20 1768 1774 10.1016/j.cub.2003.09.035 14561401

[B27] Juarez MT Kui JS Thomas J Heller BA Timmermans MCP microRNA-mediated repression of rolled leaf1 specifies maize leaf polarity Nature 2004 14 6978 84 88 10.1038/nature02363 14999285

[B28] Jones-Rhoades MW Bartel DP Computational identification of plant MicroRNAs and their targets, including a stress-induced miRNA Mol Cell 2004 14 6 787 799 10.1016/j.molcel.2004.05.027 15200956

[B29] Eckardt NA MicroRNAs regulate auxin homeostasis and plant development Plant cell 2005 14 5 1335 1338 10.1105/tpc.105.033159

[B30] Sunkar R Kapoor A Zhu JK Posttranscriptional induction of two Cu/Zn superoxide dismutase genes in *Arabidopsis* is mediated by downregulation of miR398 and important for oxidative stress tolerance Plant cell 2006 14 8 2051 2065 10.1105/tpc.106.041673 16861386 PMC1533975

[B31] Romanel E Silva TF Corrêa RL Farinelli L Hawkins JS Schrago CE Vaslin MF Global alteration of microRNAs and transposon-derived small RNAs in cotton (*Gossypium hirsutum*) during cotton leafroll dwarf polerovirus (CLRDV) infection Plant Mol Biol 2012 14 443 460 10.1007/s11103-012-9959-1 22987114

[B32] Pang MX Xing CZ Adams N Rodriguez-Uribe L Hughs SE Hanson SF Zhang JF Comparative expression of miRNA genes and miRNA-based AFLP marker analysis in cultivated tetraploid cottons J Plant Physiol 2011 14 8 824 830 10.1016/j.jplph.2010.10.006 21134704

[B33] Li G Quiros CF Sequence-related amplified polymorphism (SRAP), a new marker system based on a simple PCR reaction: its application to mapping and gene tagging in *Brassica* Theor Appl Genet 2001 14 455 461 10.1007/s001220100570

[B34] Hu JG Vick BA Target region amplification polymorphism: a novel marker technique for plant genotyping Plant Mol Bioly Rep 2003 14 289 294 10.1007/BF02772804

[B35] Liu CX Lin ZX Zhang XL Unbiased genomic distribution of genes related to cell morphogenesis in cotton by chromosome mapping Plant Cell Tiss Org 2012 14 529 534 10.1007/s11240-011-0059-8

[B36] Allen E Xie Z Gustafson AM Sung GH Spatafora JW Carrington JC Evolution of microRNA genes by inverted duplication of target gene sequences in *Arabidopsis thaliana* Nat Genet 2004 14 12 1282 1290 10.1038/ng1478 15565108

[B37] Zhang BH Pan XP Wang QL Cobb GP Anderson TA Identification and characterization of new plant microRNAs using EST analysis Cell Res 2005 14 5 336 360 10.1038/sj.cr.7290302 15916721

[B38] Qiu CX Xie FL Zhu YY Guo K Huang SQ Nie L Yang ZM Computational identification of microRNAs and their targets in *Gossypium hirsutum* expressed sequence tags Gene 2007 14 1–2 49 61 17408884 10.1016/j.gene.2007.01.034

[B39] Zhang B Wang Q Wang K Pan X Liu F Guo T Cobb GP Anderson TA Identification of cotton microRNAs and their targets Gene 2007 14 1–2 26 37 17574351 10.1016/j.gene.2007.03.020

[B40] Khan Barozai MY Irfan M Yousaf R Ali I Qaisar U Maqbool A Zahoor M Rashid B Hussnain T Riazuddin S Identification of micro-RNAs in cotton Plant Physiol Bioch 2008 14 8–9 739 751 10.1016/j.plaphy.2008.05.009 18603441

[B41] Kwak PB Wang QQ Chen XS Qiu CC Yang ZM Enrichment of a set of microRNAs during the cotton fiber development BMC Genomics 2009 14 1 457 10.1186/1471-2164-10-457 19788742 PMC2760587

[B42] Pang M Woodward AW Agarwal V Guan X Ha M Ramachandran V Chen X Triplett BA Stelly DM Chen ZJ Genome-wide analysis reveals rapid and dynamic changes in miRNA and siRNA sequence and expression during ovule and fiber development in allotetraploid cotton (*Gossypium hirsutum* L) Genome Biol 2009 14 11 R122 10.1186/gb-2009-10-11-r122 19889219 PMC3091316

[B43] Ruan MB Zhao YT Meng ZH Wang XJ Yang WC Conserved miRNA analysis in *Gossypium hirsutum* through small RNA sequencing Genomics 2009 14 263 268.44 10.1016/j.ygeno.2009.07.002 19628031

[B44] Lin ZX Zhang YX Zhang XL Guo XP A high-density integrative linkage map for *Gossypium hirsutum* Euphytica 2008 14 1 35 45

[B45] Zhang YX Lin ZX Xia QZ Zhang MJ Zhang XL Characteristics and analysis of simple sequence repeats in the cotton genome based on a linkage map constructed from a BC_1_ population between *Gossypium hirsutum* and *G. barbadense* Genome 2008 14 7 534 546 10.1139/G08-033 18545277

[B46] Yu Y Yuan DJ Liang SG Li XM Wang XQ Lin ZX Zhang XL Genome structure of cotton revealed by a genome-wide SSR genetic map constructed from a BC_1_ population between *gossypium hirsutum* and *G. barbadense* BMC Genomics 2011 14 15 10.1186/1471-2164-12-15 21214949 PMC3031231

[B47] Stam P Construction of integrated genetic linkage maps by means of a new computer package: join map Plant J 1993 14 739 744 10.1111/j.1365-313X.1993.00739.x

[B48] Kosambi DD The estimation of map distance from recombination values Ann Eugen 1944 14 172 175

[B49] Voorrips RE MapChart: software for the graphical presentation of linkage maps and QTLs J Hered 2002 14 1 77 78 10.1093/jhered/93.1.77 12011185

[B50] Munis MFH Tu LL Deng FL Tan JF Xu L Xu SC Long L Zhang XL A thaumatin-like protein gene involved in cotton fiber secondary cell wall development enhances resistance against *Verticillium dahliae* and other stresses in transgenic *tobacco* Biochem Bioph Res Co 2010 14 1 38 44 10.1016/j.bbrc.2010.01.069 20097164

[B51] Tu LL Zhang XL Liu DQ Jin SX Cao JL Zhu LF Deng FL Tan JF Zhang CB Suitable internal control genes for qRT-PCR normalization in cotton fiber development and somatic embryogenesis Chinese Sci Bull 2007 14 3110 3117 10.1007/s11434-007-0461-0

