# Supplementary figures and images for: Linkage mapping and expression analysis of miRNAs and their target genes during fiber development in cotton
Source: BMC Genomics. 2013 Oct 16;14:706. doi: 10.1186/1471-2164-14-706 (PMC4007520; doi:10.1186/1471-2164-14-706)

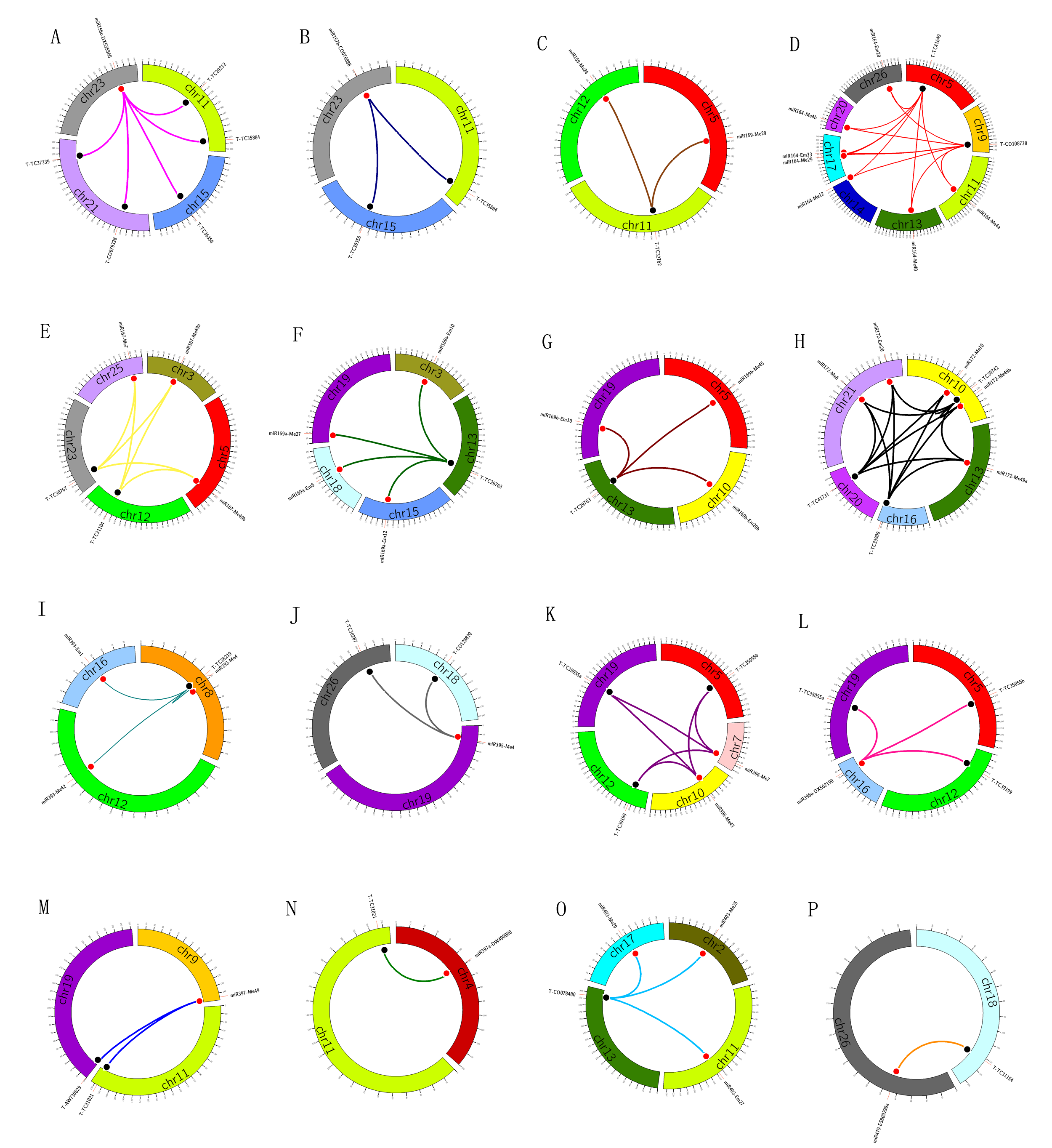

Supplement: Additional file 7: Figure S2 — Network diagram revealed by relationships between one miRNA family and their target genes. Families are as follows: A: miR156c family; B: miR157b family; C: miR159 family; D: miR164 family; E: miR167 family; F: miR169a family; G: miR169b family; H: miR172 family; I: miR393 family; J: miR395 family; K: miR396 family; L: miR396a family; M: miR397 family; N: miR397a family; O: miR403 family; P: miR479 family. Red circle: MiRNAs; black circle: Target genes. The scale marked on each chromosome represented a genetic map distance (cM). [file 1471-2164-14-706-S7.tiff]

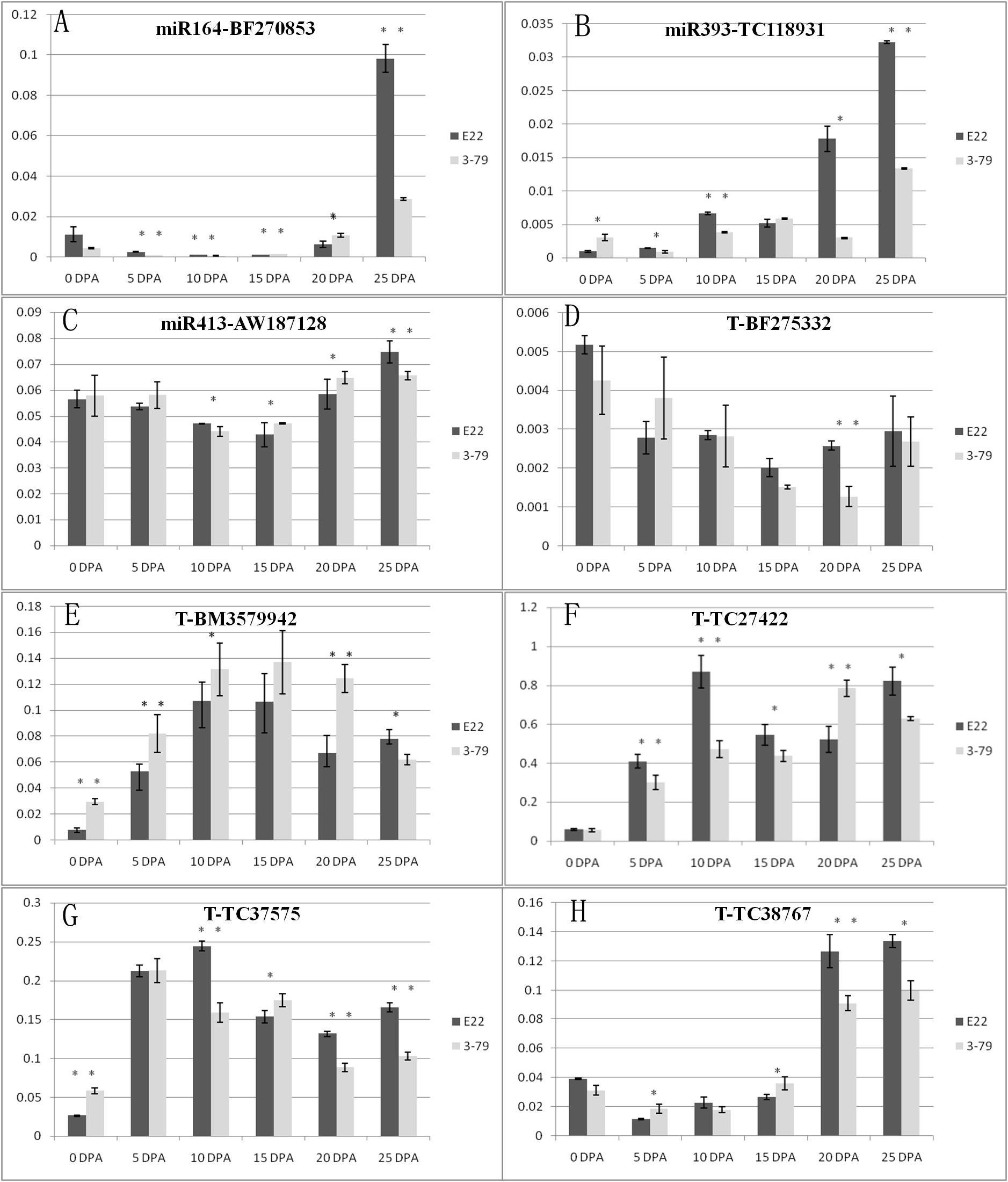

Supplement: Additional file 9: Figure S3 — qRT-PCR analysis of eight primers randomly chosen from pre-miRNAs and targets. Expression levels of Emian22 and 3–79 are shown. “*” represents P⩽0.05, and “**” represents P⩽0.01. Primers are as follows: A: miR164-BF270853; B: miR393-TC118931; C: miR413-AW187128; D: T-BF275332; E: T-BM3579942; F: T-TC27422; G: T-TC37575; H: T-TC38767. [file 1471-2164-14-706-S9.tiff]

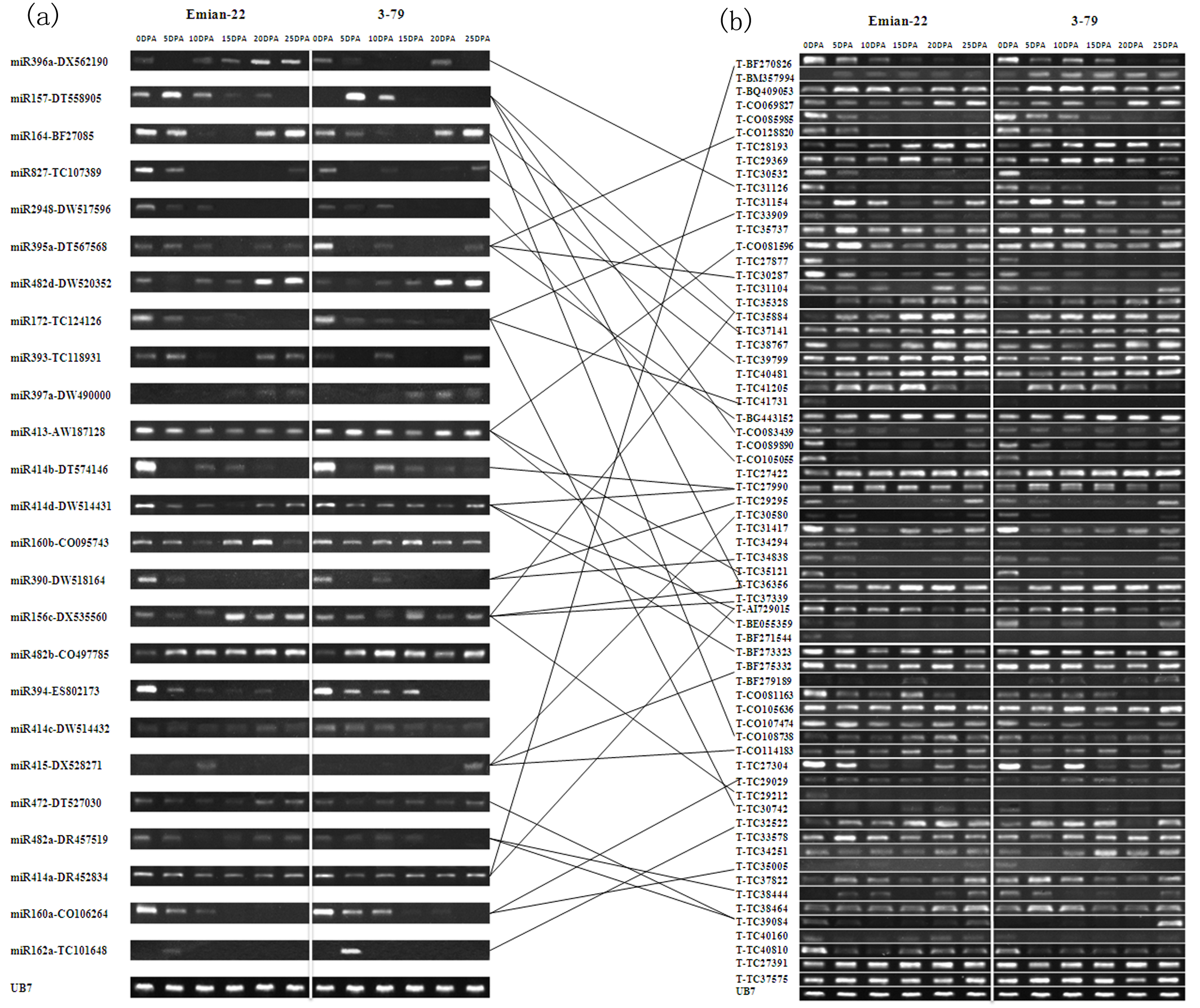

Supplement: Additional file 10: Figure S4 — Wiring diagram of target gene primers and their corresponding miRNA families. (a) RT-PCR analysis of miRNA genes; (b) RT-PCR analysis of target genes. [file 1471-2164-14-706-S10.tiff]
